# Local release of rapamycin by microparticles delays islet rejection within the anterior chamber of the eye

**DOI:** 10.1038/s41598-019-40404-0

**Published:** 2019-03-08

**Authors:** Yanliang Fan, Xiaofeng Zheng, Yusuf Ali, Per-Olof Berggren, Say Chye Joachim Loo

**Affiliations:** 1 Nanyang Institute of Technology in Health & Medicine, Interdisciplinary Graduate School, Nanyang Technological University, S639798 Singapore, Singapore; 2 School of Material Science and Engineering, Nanyang Technological University, S639798 Singapore, Singapore; 3 Lee Kong Chian School of Medicine, Nanyang Technological University, S639798 Singapore, Singapore; 4 Singapore Eye Research Institute, The Academia, S169856 Singapore, Singapore; 5 The Rolf Luft Research Center for Diabetes and Endocrinology, Karolinska Institutet, Karolinska University Hospital, SE17176 Stockholm, Sweden; 6 Singapore Centre for Environmental Life Sciences Engineering, S639798 Singapore, Singapore

## Abstract

The anterior chamber of the eye (ACE) has emerged as a promising clinical islet transplantation site because of its multiple advantages over the conventional intra-hepatic portal site. This includes reduced surgical invasiveness and increased islet graft survival rate. It also allows for enhanced accessibility and monitoring of the islets. Although the ACE is initially an immuno-privileged site, this privilege is disrupted once the islet grafts are re-vascularized. Given that the ACE is a confined space, achieving graft immune tolerance through local immunosuppressive drug delivery is therefore feasible. Here, we show that islet rejection in the ACE of mice can be significantly suppressed through local delivery of rapamycin by carefully designed sustained-release microparticles. In this 30-day study, allogeneic islet grafts with blank microparticles were completely rejected 18 days post-transplantation into mice. Importantly, allogeneic islet grafts co-injected with rapamycin releasing microparticles into a different eye of the same recipient were preserved much longer, with some grafts surviving for more than 30 days. Hence, islet allograft survival was enhanced by a localized and prolonged delivery of an immunosuppressive drug. We envisage that this procedure will relieve diabetic transplant recipients from harsh systemic immune suppression, while achieving improved glycemic control and reduced insulin dependence.

## Introduction

Diabetes is a debilitating disease with high morbidity^1^. Poor glycemic control results in long-term complications such as blood vessel damage, nerve damage, kidney failure and cardiovascular disease^2^. Diabetes stems from the inability of the body to supply sufficient insulin to meet the metabolic demands. As a result, exogenous insulin supplementation is required for Type-1 diabetic and ‘C-peptide low’ Type-2 diabetic patients to maintain glucose homeostasis. Nonetheless, insulin therapy has life-threatening side-effects, such as acute hypoglycemia risk and chronic unnatural fluctuations in blood glucose levels^3^. As such, islet transplantation has emerged as a promising treatment for diabetes as it overcomes the deficient or inadequate insulin secretion in the most natural way and without any of the negative side-effects seen with exogenous insulin therapy^4–6^.

Islet transplantation is routinely done in many diabetic centers^7^ applying protocols where islets are injected through the hepatic portal vein to the liver^4,8^. However, low oxygen tension, sheer physical stress within the hepatic portal system and the induction of inflammation lead to significant islet graft dysfunction and loss^9–12^. To compensate for this loss, a large number of islets are transplanted into a patient. The current accepted standard of islet transplantation is a minimum of 5000 isolated islet equivalents per kilogram (IEQs/kg) body weight to relieve insulin dependence^8^. To achieve this number, multiple donors may be required to treat a single patient, which inadvertently strains donor adequacy. The anterior chamber of the eye (ACE), as a novel site for islet transplantation, has unique advantages over the currently used hepatic portal system. The ACE is a less invasive and conducive transplant site compared to the hepatic portal system^13^. The iris has a high concentration of blood vessels that enables rapid vascularization of the grafted islets, minimizing islet hypoxia and death. Besides, a transparent cornea allows for easy access to the islet grafts thereby enabling non-invasive, longitudinal imaging of the islet grafts at single-cell resolution^14^. Islets transplanted into the ACE have been reported to functionally mirror islets within the pancreas^15^. Furthermore, islet grafts at this site have been shown to significantly reduce insulin dependence in diabetic non-human primates^13^. Islets transplanted into the ACE have also been used to study *in vivo* immune responses in an allogeneic setting, in real-time (islets from DBA/2 mice into C57BL6/J recipients)^16^.

Clinical pancreatic islet transplantation is limited by donor scarcity and recipient compatibility. A recipients’ ability to be on lifelong systemic immunosuppression is a key determinant for transplant suitability. As such, islet transplantation may be preferred for patients who have already received, or who are concurrently receiving, a kidney transplant^7^. Immunosuppressive drugs circulate throughout the body to dampen the immune system. While it works to prevent graft immune-rejection, its systemic circulation inadvertently renders the host vulnerable to a myriad of common infections. In addition, the presence of immunosuppressive agents in off-target tissues results in undesirable side-effects such as peripheral oedema and kidney dysfunction^17–20^. To reduce patient burden to drug-load and undesirable side-effects, immunosuppressive drug delivery needs to be better engineered for targeted and tunable exposure. Here, a further advantage of transplanting islets into the ACE is that the relatively enclosed environment provides an opportunity for localised immunosuppression. Rodent studies have shown that an immune response against allogeneic islets transplanted into the ACE starts approximately 7 days post-transplantation following islet vascularization^16^. By 14 to 21 days post-transplantation, the islet grafts are completely rejected in the absence of immunosuppression. To keep the grafts from being rejected by the host, lifelong systemic administration of immunosuppressive agents is therefore required.

Biodegradable microparticle drug delivery systems can be fabricated to encapsulate various drugs, and tuning them for controlled release^21–23^. Controlled drug release is superior because customizable drug exposure in terms of concentration, localization and duration maximize drug efficacy and mitigate unwanted fluctuations in drug dosage^24,25^. By exploiting engineered particulate drug delivery systems for islets transplanted into the ACE, we have the ability to circumvent current clinical challenges of long-term immunosuppression and low islet functional efficacy. Engineered particulate drug delivery systems can be tailor made, are both easy-to-inject and biodegradable, posing minimal harm and offering greater convenience to islet graft recipients. Hence, we hypothesize that a novel engineered sustained-releasing immunosuppressive microparticle system can be co-transplanted together with islets into the ACE, to prevent graft-host immune rejection in a localized milieu.

We now report the development of a novel sustained-releasing microparticle formulation with an effective dose of rapamycin over 30 days. Our *in vitro* data show that this microparticle formulation is safe and does not impede islet function. When co-transplanted together with rodent islets into the ACE of an allogeneic recipient, these microparticles significantly delayed graft rejection *in vivo*. Furthermore, the rapamycin dose achieved locally through our microparticle formulation was found to be significantly lower as compared to the current systemically-delivered clinical dose. Yet, the therapeutic effect was comparable. Our results thus suggest that local delivery of immunosuppressive agents through bioengineered microparticles is an effective strategy to prevent islet graft rejection, while mitigating the hazardous side effects of such biologically harsh agents when delivered systemically.

## Materials and Methods

Poly(l-lactide-co-glycolide) (PLGA) 50:50 (IV 2.0), polycaprolactone (PCL) (MW 10 kDa) and poly(vinyl alcohol) (PVA) (MW 30–70 kDa) were obtained from Sigma-Aldrich (Singapore). Rapamycin from *Streptomyces hygroscopicus* was obtained from Apollo Scientific (UK). High performance liquid chromatography (HPLC) column BC-Poroshell 120 (EC-C18, 4.6 × 100 mm, 2.7 μm) was obtained from Agilent (Singapore). HPLC grade methanol (MEOH), dichloromethane (DCM) and acetonitrile were from Tedia (US). Dulbecco’s Phosphate Buffer Saline (DPBS) of pH 7.4 was obtained from Life Technology (Singapore). Purified water was obtained from Milli-Q deionized H2O (Biocel Ltd.). AMO Endosol Balanced Salt Solution for Ophthalmic Irrigation was purchased from Abbott (US). Anterior chamber cannula (angled 45°, 0.50 × 22 mm, 25 G) and lacrimal cannula (curved, 0.45 × 28 mm, 26 G) were purchased from Beaver-Visitec International (MA, US). Hamilton GASTIGHT® Syringes (100 µL, Model 1710, PTFE Luer Lock) were purchased from Hamilton Robotics. Viscotears® liquid gel was purchased from Alcon (Novatis). All items were used as received.

### Microparticle fabrication, characterization and *in vitro* release

Particle fabrication was based on oil-in-water emulsion solvent evaporation method^26^. 0.3 g of polymer as stated above was dissolved with rapamycin (1.33% w/w) in 3 ml DCM homogenously, then the polymer solution was poured into a deionized (DI) water with PVA (0.5% w/v) and emulsified at 400 rpm using an overhead stirrer (Calframo BDC1850-220) at ambient temperature for three hours. After DCM evaporation, the as-formed particles were collected and washed with DI water. Sampled particles were lyophilized and stored in a freezer. For characterization, particles were imaged under the JEOL JSM-6360A Scanning Electron Microscope (5 kV) for surface morphology and cross-section. Samples were prepared and quantified as previously described^26^. 4 mg of particles were weighed and dispersed in a release medium (10 ml DPBS buffer). All samples were maintained in a 37 °C shaking incubator (orbital shaker incubator, model: LM-570RD). Release medium was collected daily up to 30 days. Encapsulation efficiency (EE) is the percentage of drug encapsulated over the total amount of drug added. Approximately 5 mg of particles were weighed and dissolved in DCM (1 ml). Methanol (5 ml) was then added to precipitate out the polymer. Polymer precipitates were centrifuged and the supernatant was drawn for HPLC analysis. HPLC analysis for drug content was done as previously described^26^ for *in vitro* daily release and EE calculation.

### Islets isolation

We purchased C57BL/6 J and DBA/2 mice from the Jackson Laboratories (Bar Harbor, ME). All experiments were approved by the local animal ethics committees at SingHealth Academia and SingHealth Institutional Animal Care and Use Committee (IACUC). All experiments were performed in accordance to IACUC Protocol #; 2013SHS/816, that is in accordance to relevant guidelines and regulations. Islet donors (DBA/2, males) were sacrificed at 16 weeks of age. Islet recipients (C57BL/6 J, males) were transplanted at 12 weeks of age.

Murine islets were isolated as described previously^27^. Briefly, mice were anesthetized and killed. The abdomen was opened, and with the pancreas exposed an enzyme solution of Collagenase type V (Sigma) at a final concentration of 0.5 mg/ml in Hank’s Balanced Salt Solution (HBSS) was injected through the main bile duct until full distension was achieved. The pancreatic tissue was then surgically removed and immersed in the enzyme solution. Digestion was performed in a 5-6 minutes incubation at 37 °C, with gentle shaking, after which enzyme kinetics were effectively slowed down by the addition of cold HBSS supplemented with 10% fetal bovine serum (FBS). Mechanical disruption of the digested pancreatic tissue was achieved by repeated passages through 14 G needles, and tissue was then filtered through a 450-µm metal mesh. Islet purification was obtained by centrifugation at 676 g for 15 min on discontinuous Euro-Ficoll gradients, providing an islets of purity >90%.

Before transplantation, islets were cultured overnight at 37 °C, 5% CO_2_, in Connaught Medical Research Laboratories (CMRL) medium supplemented with 10% fetal calf serum, 2 mmol/l l-glutamine, 100 µg/ml penicilin streptomycin, and 25 mmol/l HEPES buffer (CMRL-10).

### Islet Glucose Stimulated Insulin Secretion (GSIS) Assay

Aliquots of freshly isolated murine islets were cultured in 35-cm tissue culture dishes, containing CMRL-10 (2 ml) in the absence and presence of rapamycin-loaded microparticles, or blank microparticles or rapamycin (20 nM). After 24 hr culture, islets were removed and washed twice in RPMI medium. Three to five mouse islets per dish were picked into a 12-well plate, and starved in 1 ml of low-glucose buffer, containing glucose (3 mM), NaCl (125 mM), KCl (5.9 mM), CaCl_2_ (2.56 mM), MgCl_2_ (1.2 mM), HEPES (25 mM) and 0.1% (wt%) BSA (Ca-10 buffer), at 37 °C for 1.5–2 hr. After starvation, the islets were picked and incubated with fresh 0.5 ml low-glucose buffer for 30 min. Next, islets were removed and washed 3 times with 1 ml fresh low-glucose buffer. Islets were then transferred to 0.5 ml high-glucose buffer containing glucose (16 mM) in Ca-10 buffer and incubated for 30 min. Thereafter islets were removed and lysed with RIPA Lysis buffer (ThermoFisher). Supernatants after 30 min incubation with low-glucose as well as high-glucose buffer were collected for insulin quantification by Mouse Insulin ELISA (Mercodia), normalized to the number of islets.

### Transplantation of pancreatic islets and particles to the ACE

Particles were assembled into required amounts and sterilized under UV lamp (UVP, Thermo Scientific) at 252 nm for 20-30 min and particles were stored at -20 °C until they were used. Immediately before transplantation, islets were handpicked under the microscope and divided in aliquots of 10-50 islets per recipient. The mouse was first put under general anesthesia induced by 2% isoflurane mixture inhalation (isoflurane, Baxter, IL, USA). Then islets were transferred from culture media to sterile PBS and were aspirated into a blunt anterior chamber cannula connected to a 1-ml Hamilton syringe (Hamilton) via 0.4-mm polythene tubing (Portex Limited). To obtain post-operative analgesia, TobraDex (Tobramycin, Dexamethasone Ointment, Alcon) was applied on the eye. Under a stereomicroscope, we punctured the cornea close to the sclera at the bottom part of the eye with a 27-gauge needle and took great care not to damage the iris and to avoid bleeding. Next, particles, which were pre-suspended in 5% BSA in PBS buffer (filtered), were aspirated using lacrimal cannula. We gently inserted this cannula and slowly injected the particles into the ACE, where they settled onto the iris. Islets were then injected in a similar manner through the same opening using the anterior chamber cannula. After injection, we carefully withdrew the cannula and left the mouse lying on its side. The mice quickly recovered and showed no signs of stress or irritation from the manipulated eye.

### *In vivo* imaging of islets transplanted to the ACE

At the indicated time points after transplantation, mice were anesthetized with a 30% oxygen and a ~2% isoflurane mixture and placed on a heating pad. We restrained the mouse head with a stereotaxic head-holder (SG-4N, Narishige) and positioned the eye containing the engrafted islets facing upwards. The eyelid was carefully pulled back to hold the eye gently at the coreneoscleral junction with a pair of tweezers attached to a UST-2 Solid Universal Joint (Narishige). The tips of the tweezers were covered with a single piece of polythene tubing, creating a loop between the two tips. This arrangement permitted a flexible but stable fixation of the head and eye without causing damage or disrupting the blood circulation in the eye. Imaging was performed using an upright Leica TCS SP8 DM6000 CFS confocal microscope (Leica Microsystems) equipped with White Light Laser (470–670 nm) using a long-distance water dipping lens (Leica HXC APO 10 × 0.3 w)^28^. Viscotears (Novartis) was used as an immersion medium between the lens and the mouse eye. Backscatter signal imaging of each islet was obtained using a 633 nm laser beam as previously described^29^. To visualize the blood vessels, the animal was injected with 150 kDa Fluorescein isothiocyanate (FITC)-labeled dextran (20 mg/kg, Sigma) via the retro-orbital venous sinus. Fluorescence emission from FITC was obtained by excitation at 488 nm and detection between 500 and 550 nm. No signs of photo-damage in islet cells were observed. Leica Confocal Software (version 2.61), and ImageJ were used to process images.

### Islet rejection and survival measurements

The 3D volume of islets was measured based on confocal images taken at time points using ImageJ^30,31^. Briefly, the images were filtered using the 3D median method with proper radius. The islet object was segmented from the filtered image using a carefully set threshold. The volume was calculated based on the segmented image over multiple Z-stacks at a step size of 3 µm. Volume measurements of day 3 were set as the maximum volume and were used to normalize the volume change.

### Statistical analysis

For individual experiments, the number of animals or islets used (n) is included in each figure legend in parenthesis. All results are expressed as mean ± sem. Statistical analysis were performed with GraphPad Prism 7 and Microsoft Excel 2016. Student’s t-test was used unless otherwise reported in the figure legend. P values < 0.05 were considered as statistically significant.

## Results

### Microparticles can be tuned to release an effective dose of rapamycin *in vitro*

**Figure 1:**
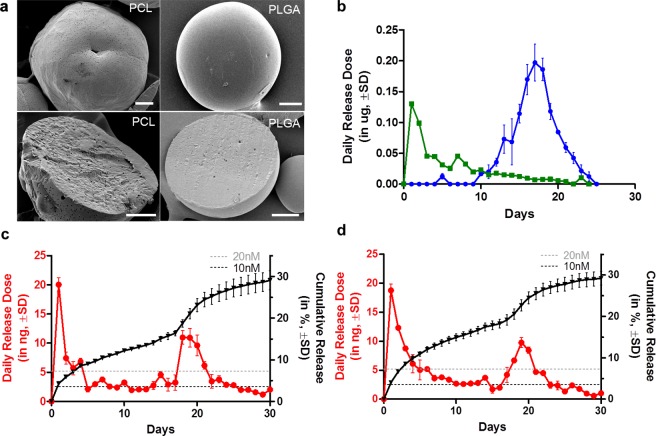
Rapamycin microparticles and their release under *in vitro* condition. Surface morphology and cross-sectional views of rapamycin in PCL microparticle (**a**, left column) and rapamycin in PLGA microparticle (**a**, right column) under scanning electron microscope (SEM). (**b**) shows daily released rapamycin amounts measured from 50 microparticles (PCL () released drug immediately while PLGA () had 10 days delay in release onset), a mixture of these two microparticles at 1:1 ratio showed a sustained release over 30 days **(▼)** at a higher-than-20 nM daily dose () (mean ± SD, n = 3) in phosphate buffer (**c**) and balanced salt buffer (**d**). Daily release rate was scaled down from release study performed using 5 mg of microparticles due to practicality. Scale bar: 20 μm.

Two controlled-releasing particulate delivery systems were developed with different biodegradable polyesters that are FDA-approved, i.e. PLGA and PCL. These polymers were chosen primarily because of their ability to be tuned for controlled release of rapamycin. The low bioavailability of unstable rapamycin also warrants it to be protected and delivered through a specific delivery system^26^. Both rapamycin-encapsulated microparticles were prepared using a facile oil-in-water emulsion solvent evaporation method. Figure 1a shows the scanning electron microscopy micrographs of the microparticles. For PCL microparticles, they were observed to be porous, with minute pores distributed throughout the structure of the particle (Fig. 1a). We postulate that the porous morphology as such would facilitate water diffusion and accelerate drug release. *In vitro* drug release in phosphate buffer saline (PBS) solution (Fig. 1b) showed rapamycin being released through a diffusion controlled manner, with a rate constant of 0.7176 (R^2^ = 0.97)^26^. Beyond day 10, the amount of rapamycin released was dramatically reduced with no drug detected at day 30. Rapamycin-loaded PLGA microparticles, on the other hand, possessed a dense and significantly less porous morphology (Fig. 1a), with rapamycin release commencing only at day 10, i.e. a lag-phase release. Release profile from PLGA microparticles followed that of the Hoffenberg release model (R^2^ = 0.99)^32,33^, peaking at day 18 and diminishing at day 30 (Fig. 1b). Based on previous studies, the targeted rapamycin dosage should lie between 10 to 20 nM per day (i.e. 2.6 to 5.2 ng/day, calculated according to the description given in Supplementary Table S1)^34,35^. In a prior study^14^, islet re-vascularization occurs at 3-6 days post transplantation in the eye, and the allogeneic islet rejection only happens upon vascularization. Since the allogeneic islet rejection only occurs when the islets become vascularized, the immunosuppressive treatment has to start as early as 3 days post transplantation, and its targeted dose has to be maintained above the working concentration for a sustained duration. As neither of the individual microparticle delivery systems on their own could achieve this targeted dose throughout the 30 day period, a combination of these two systems was required to provide an extended release of bioactive rapamycin.

A mixture of both PCL and PLGA microparticles at a ratio of 1:1 was evaluated for rapamycin release in PBS buffer (Fig. 1c). The release profile of the microparticle mixture displayed two peaks at day 0 and day 18, which corresponds to the maximum daily release rate observed in their respective individual drug release profiles (Fig. 1b). To better mimic the ACE environment, we repeated the release study in balanced salt solution (BSS), an intraocular irrigation solution with a composition similar to that of aqueous humor^36^. The *in vitro* result similarly confirmed that the daily rapamycin dosage requirement could be achieved. Hence, this delivery system meets the daily minimum effective dosage of rapamycin throughout the 30 day period and could thus be applied in our *in vivo* studies.

**Figure 2:**
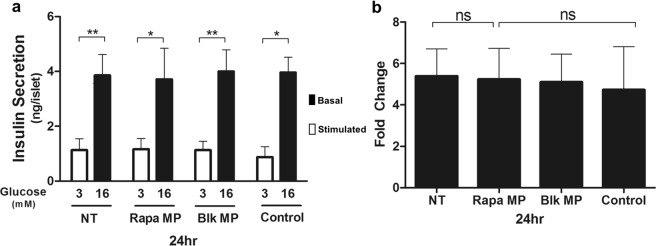
Effect of rapamycin microparticles and blank microparticles on *in vitro* glucose-stimulated insulin secretion (GSIS) from islets. (**a**) Aliquots of 3-5 hand-picked islets were cultured in RPMI-1640 medium in the absence of rapamycin (NT) and in the presence of 20 nM rapamycin microparticles (Rapa MP), blank microparticles (Blk MP) and 20 nM free drug (Control) for 24 h, followed by shifting glucose concentration of culture media from 3 mM to 16 mM. Thirty minutes later, aliquots (100 μl) of media were sampled for insulin measurements using ELISA. *P < 0.05; **P < 0.01; □, basal; ■, stimulated. (**b**) Fold change in insulin release was compared across the different treatments stated in A). Difference measured was not significant (ns). Bars indicate means ± SEM.

Previous studies have suggested that rapamycin may affect islet function^37,38^. To evaluate the effect of released rapamycin on islet function, glucose-stimulated insulin secretion (GSIS) of islets was conducted *in vitro* (Fig. 2). Here, islets were co-incubated with the rapamycin-loaded microparticle delivery system (at 1:1 ratio), non-drug-loaded microparticle mixture or the equivalent amount of free drug for 24 h before GSIS. The results show that GSIS from islets treated with blank microparticles was not significantly different from the non-treated islets (NT), and the free rapamycin (Control) or rapamycin microparticle delivery system did not reduce the fold change in terms of glucose stimulation index. This suggests that the released rapamycin did not compromise islet function.

### Rapamycin microparticles delay islet graft immune rejection *in vivo*

To test the hypothesis that sustained-releasing rapamycin-loaded microparticles are able to suppress islet rejection *in vivo*, these microparticles were co-transplanted with islets into the right ACE of an allogeneic recipient mouse. Concurrently, blank, control microparticles were co-transplanted with a similar number of islets, obtained from the same donor mouse, into the left ACE of the recipient mouse. Both transplanted islets and microparticles were longitudinally monitored for up to 40 days by established bright-field as well as confocal microscopy^16^.

**Figure 3:**
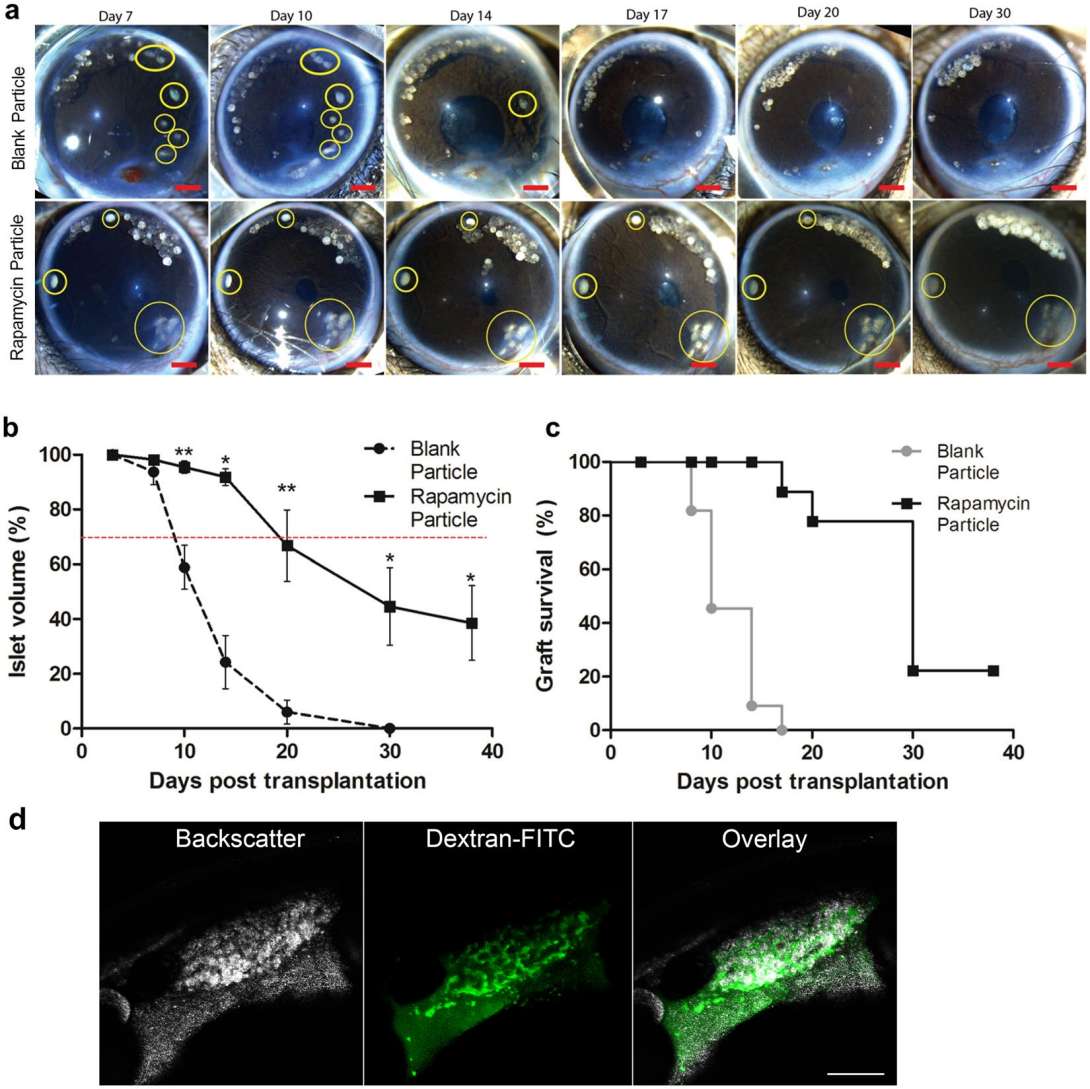
*In vivo* imaging of transplanted islets in the presence of rapamycin and blank microparticles. (**a**) Images of mouse eyes transplanted with allogeneic islets (yellow circle) and blank microparticles (first row) or rapamycin microparticles (second row). With the blank microparticles, visible islets decreased gradually during the first 14 days and disappeared fully on day 17. In the eye transplanted with rapamycin-loaded microparticles, the grafted islets were present throughout the 30 days, without any visible decrease in islet volume. Scale bar: 500 µm. (**b**) Changes in the average islet volume with blank particles (●) vs. the islet volume with rapamycin microparticles (■). The onset of islet rejection is marketed by the red dotted line at the 70% of initial islet volume. Data based on 5–9 islets/time point from 5 eyes with rapamycin microparticles and 3-10 islets/time point from 3 eyes with blank microparticles. Results presented as means ± SEM; *P < 0.05; **P < 0.01. (**c**) Survival curves of islet grafts in the ACE based on volume (Blank microparticle: n = 9; Rapamycin microparticle: n = 9). (**d**) *In vivo* imaging of the islets co-transplanted with rapamycin microparticles at day 30 post transplantation by confocal microscopy. Vasculature was visualized by retro-orbital injection of dextran-FITC prior to imaging. Scale bar = 100 µm.

**Figure 4:**
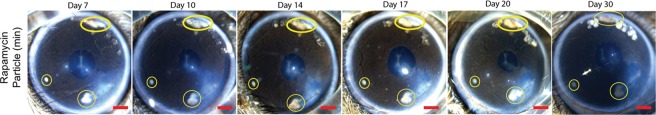
Effects of microparticle number on islet rejection. The minimum number of particles transplanted was 20. The 3 islets transplanted were present throughout the 30 days of monitoring, which suggests islet rejection can be delayed with even a small amount of particles.

In the absence of rapamycin, the islet number rapidly decreased within the first 14 days of transplantation (Fig. 3a, first row). By post-transplantation Day 17, all transplanted islets in the left ACE (blank microparticles) visually disappeared, suggesting complete islet immune rejection. In comparison, islets within the ACE of the right eye (rapamycin-releasing microparticles) remained up to post-transplantation Day 30 (Fig. 3a, second row). The data suggests that rapamycin-releasing microparticles are able to protect grafted islets from immune rejection in a localized milieu of the ACE. In addition, we reduced the number of rapamycin particles transplanted into the ACE in one mouse to test whether islet rejection rate would change when the ratio of particle-over-islets was reduced from 10 to approximately 4 (Fig. 4). *In vivo* observation showed no difference in islet rejection rate when this ratio was decreased (Fig. 3b), suggesting that the effective microparticle to islet ratio can be further reduced without compromising islet survival. Over time, the microparticles turned white and opaque, suggesting that they were degraded^39^.

Next, by quantifying changes in islet volume, we were able to determine the relative rates of islet rejection. When the islet size is reduced to ≥ 30% of the original imaged size (at post-transplantation Day 3), the islets are arbitrarily considered as fully rejected^16^. The volume of islets in the left ACE, containing blank microparticles, decreased by approximately 30% between post-transplantation Day 7 and Day 10 (Fig. 3b). By post-transplantation Day 14, the islet volume remaining in the left ACE was only 20% when compared to the initial time point. This further corroborates previous evaluation of *in vivo* islet graft allogeneic rejection rates across a 40-day period^16^.

In contrast, islets with rapamycin-loaded microparticles had a volume of more than 70% between post-transplantation Day 3 and Day 20 (Fig. 3b). The difference in islet volume between blank and rapamycin microparticles was seen as early as Day 3 of post-transplantation. The rate of islet volume loss after post-transplantation Day 20 was significantly lower for the ACE with rapamycin-loaded microparticles compared with blank microparticles (Fig. 3b). Hence, the preservation of islets with the rapamycin microparticles was evident. We further validated this observation with survival curves based on numbers of islets rejected (Fig. 3c). The islets with rapamycin microparticles survived longer than the islets with the blank microparticles. The condition and survivability of transplanted islets were assessed *in vivo* longitudinally across time, instead of using destructive, end-point histological assessments. We show that the islets co-transplanted with rapamycin particles are fully vascularized at post transplantation Day 30 (Fig. 3d), using FITC-dextran injected into retro-orbital sinus, thus excluding a possible negative effect of rapamycin on islet revascularization. Furthermore, the condition of islets was measured *in vivo* using reflected light (backscatter) which acts as a surrogate for hormone-containing large dense core vesicles within cells of the islet^29^. Taken together, the rapamycin microparticles significantly delayed islet rejection when co-transplanted into the ACE.

## Discussion

The high risk of systemic immunosuppression is one of the key limitations of organ transplantations, especially for islet transplantation. The negative side-effects of systemic immunosuppression therapy include, but are not limited to, the high dosages required to suppress whole-body immune response. Hence, the use of immunosuppressive drugs at high dosages predisposes patients to unnecessary risks such as infectious disease and cancer, thereby compromising quality of life and increasing the risks of mortality^40^. Local immunosuppression is therefore preferred as it protects the transplanted organ on site against the host immune system at lower drug dosages, while minimizing the risks and side-effects of systemic immunosuppression.

Islet transplantation to the ACE is shown to have better islet vascularization and higher survival rates compared to other transplantation sites^16,41,42^. As the ACE is relatively enclosed, we tested whether islets co-transplanted with rapamycin-releasing microparticles could achieve local immunosuppression. If so, the ACE would provide numerous advantages for islet transplantation. Here, we successfully developed a sustained-releasing microparticle delivery system, for controlled rapamycin release that is above the minimum daily dosage across 30 days – our study period. As a proof, T-cell proliferation was effectively suppressed by these rapamycin-releasing microparticles over a month^26^. After transplantation into a mouse model for allogeneic islet rejection evaluation, the islets co-transplanted with rapamycin-loaded microparticles were shown to survive for a significantly longer period of time, whereas islets with blank microparticles (control) were rejected within the first two weeks. Taken together our data suggests that islets transplanted into the ACE can be locally protected through a suitable, biocompatible, sustained-releasing delivery system of rapamycin.

When determining a suitable carrier for rapamycin, microparticles were chosen because of their robustness and versatility in tuning their release kinetics and profile. Nanoscale drug carriers, on the other hand, have a higher tendency for burst release^43^ and therefore a shorter drug release duration. In addition, the optimal size of microparticles would prevent rapid loss of particles in the ACE, due to the frequent turnover of the aqueous humour. In view of these concerns, a library of different microparticles, encapsulated with rapamycin, were developed at an average size of 100 µm that would also allow for transplantation through a cannula (see Supplementary Table S2). As the selected immunosuppressive agent, rapamycin, is highly hydrophobic, water-insoluble and unstable^44^, designing a protective, sustained-releasing microparticle formulation was challenging. To achieve an immediate release of hydrophobic rapamycin from hydrophobic polyesters, low molecular weight polymers were chosen^26^. The low molecular weight, amorphous PCL gave porous microparticles (Fig. 1a) that provided an immediate release of rapamycin from day 0. By improving on drug encapsulation efficiency (95.7% and 100%), fewer microparticles were also needed to achieve the minimum required daily rapamycin dose of 20 nM. The use of PLGA, on the other hand, provided a lag-phase release that could overcome the limitation of PCL. A combination of these microparticles therefore provided sustained release of rapamycin that did not affect the insulin-secreting function of the islets (Fig. 2).

With an optimized controlled-releasing formulation, we went on to successfully transplant allogeneic islets together with microparticles into the ACE of mice. One main advantage of transplantation into the ACE is the ease of longitudinal non-invasive observations of islet grafts. This allowed for the constant monitoring of islets in the ACE and the simultaneous tracking of the microparticles. In contrast to the rapidly settling and immobile islets grafts, microparticles in the ACE were found at the periphery of the iris, with occasional shifting of the location of the microparticles. Mobility of the microparticles however did not seem to affect the visual acuity of the host mouse as observed through their behavior.

Based on a previous study of DBA/2 mouse islets in C57BL/6J mice, islet rejection occurred between day 7 and 14 whereby islet volume rapidly decreased to 50% of its original volume on day 14^16^. In addition, it has previously been shown that apoptotic islet cells are closely associated with ruffled T cells (active form of T cells) and not iris or other ocular cells^16^. During the longitudinal observation, there were no signs of damage on the iris or other ocular cells. Hence, the reported observation of islets condition and mass change have to be mainly contributed by the rapamycin versus blank microparticles and the rejection effect.

In our study, islets co-transplanted with blank microparticles showed a rejection timeframe between post-transplant day 7 and 10, corresponding to the allogeneic islet rejection model (Fig. 3a). In addition, islet volume decreased to a similar extent on post-transplant day 14 (Fig. 3b). The similar rejection rate on the islets co-transplanted with blank particles as described in prior study^16^ indicates that it is unlikely that rapamycin can be exchanged between the two eyes of the same recipient. Interestingly, islets with rapamycin-loaded microparticles showed a lower degree of volume loss and remained visually present until day 30. These above points jointly strongly suggest that rapamycin-loaded microparticles effectively suppressed the immune response and delayed islet rejection within the ACE.

Furthermore, islet survival in the ACE was not dependent on their distance from microparticles, suggesting the presence of an effective rapamycin dose within the aqueous humor. Pigmentation of microparticles occurred gradually over time during the 40 days post transplantation. Although this posed a difficulty for visually counting the microparticles, the microparticles could still be imaged through collection of backscattered light. The degradation of microparticles in the ACE is visible as the entire microparticles swells and becomes fully opaque towards the end of release period. This phenomenon was also observed in *in vitro* release studies. As the aqueous humor is constantly replenishing itself over time^45^, the degraded by-products is perhaps excreted naturally, and would not accumulate to induce pH changes affecting islet or eye function.

In this study, we have shown that islet allografts exposed to a localized, prolonged delivery of rapamycin through microparticles show considerably enhanced survival. The success of this model demonstrates the advantages of local immunosuppression and opens the possibility of transplanting islets to the ACE as a metabolic therapy for diabetes. Using this approach, the deleterious effects of chronic immunosuppression and cytotoxicity to grafted islets can be minimized, and the risk of infection post transplantation can be lowered. Through the use of a tunable sustained-releasing microparticle delivery system, release of drugs beyond 30 days is also highly feasible. In addition, the ability to load different agents into microparticles will expand the functional applicability of such a bioengineered delivery system.

## Supplementary information

supplementary information
