# Supplementary material for: Local release of rapamycin by microparticles delays islet rejection within the anterior chamber of the eye
Source: Sci Rep. 2019 Mar 8;9:3918. doi: 10.1038/s41598-019-40404-0 (PMC6408557; doi:10.1038/s41598-019-40404-0)
Supplement: Supplementary file 1 — supplementary information [file 41598_2019_40404_MOESM1_ESM.docx]

Supplementary information

Local release of rapamycin by microparticles delays islet rejection within the anterior chamber of the eye

Yanliang Fan^1,2,#^, Xiaofeng Zheng^3,4,#^, Yusuf Ali^3,4^, Per-Olof Berggren^3,4,5,*^ and Say Chye Joachim Loo^2,6,*^

^1^ Nanyang Institute of Technology in Health & Medicine, Interdisciplinary Graduate School, Nanyang Technological University, S639798, Singapore

^2^ School of Material Science and Engineering, Nanyang Technological University, S639798, Singapore

^3^ Lee Kong Chian School of Medicine, Nanyang Technological University, S639798, Singapore

^4^ Singapore Eye Research Institute, The Academia, S169856, Singapore

^5^ The Rolf Luft Research Center for Diabetes and Endocrinology, Karolinska Institutet, Karolinska University Hospital, SE17176, Stockholm, Sweden

^6^ Singapore Centre for Environmental Life Sciences Engineering, S639798, Singapore

^#^ Co-first authors

**Table S1: Mass of rapamycin calculated from mice ACE volume & target concentration of 20nM.**

| Target Concentration | Rapamycin M_w_ | Volume* per Day | Mass |
| --- | --- | --- | --- |
| 20 nM | 914.2 daltons | 5.9 µL x 2 times/hr x 24 hr = 283.2 µL | 5.2 ng |

*The volume of aqueous humor in ACE is 5.9 uL. And it replenished completely every 30-40 mins. Hence we estimated the volume of aqueous humor per hour equals to 5.9 µL x 2 times/hr.

**Table S2: Characterization of rapamycin microparticles: average diameter, drug loading and encapsulation efficiency. *Value was provided by supplier.**

| Formulation | Polymer M_w_ | Diameter ± SD (μm) | Drug Loading | Encapsulation Efficiency ± SD |
| --- | --- | --- | --- | --- |
| PLGA | 11427.5 | 87 ± 27 | 1.33% | 95.77% ± 1.15% |
| PCL | 14000* | 122 ± 28 | 1.33% | 103.73% ± 1.1% |
